# Intracranial Thrombus Morphology and Composition Undergoes Time-Dependent Changes in Acute Ischemic Stroke: A CT Densitometry Study

**DOI:** 10.3390/ijms17111959

**Published:** 2016-11-23

**Authors:** Slaven Pikija, Jozef Magdic, Vladimir Trkulja, Peter Unterkreuter, Johannes Sebastian Mutzenbach, Helmut F. Novak, Friedrich Weymayr, Larissa Hauer, Johann Sellner

**Affiliations:** 1 Department of Neurology, Christian Doppler Medical Center, Paracelsus Medical University, 5020 Salzburg, Austria; s.pikija@salk.at (S.P.); j.mutzenbach@salk.at (J.S.M.); h.novak@salk.at (H.F.N.); 2 Department of Neurology, Univerzitetni Klinični Center, 2000 Maribor, Slovenia; jozef_magdic@yahoo.com; 3 Department for Pharmacology, School of Medicine, University of Zagreb, 10000 Zagreb, Croatia; vtrkulja@mef.hr; 4 Department of Neurology, Bezirkskrankenhaus Lienz, 9900 Lienz, Austria; p.unterkreuter@kh-lienz.at; 5 Division of Neuroradiology, Christian Doppler Medical Center, Paracelsus Medical University, 5020 Salzburg, Austria; f.weymayr@salk.at; 6 Department of Psychiatry, Christian Doppler Medical Center, Paracelsus Medical University, 5020 Salzburg, Austria; l.hauer@salk.at; 7 Department of Neurology, Klinikum rechts der Isar, Technische Universität, 81675 München, Germany; 8 Institute of Linguistics, University of Salzburg, 5020 Salzburg, Austria

**Keywords:** acute ischemic stroke, intracranial clot, vascular disease, atherosclerosis, neuroimaging, hyperdense artery sign, biomarker

## Abstract

The aim of our study was to assess whether cerebral artery clots undergo time-dependent morphological and compositional changes in acute ischemic stroke. We performed a retrospective chart review of patients admitted within 5 h from symptom onset to three European stroke centers and evaluated non-contrast-enhanced CT (NECT) for hyperdense artery signs (HAS) in 2565 scans. The occlusion site, density of HAS expressed in Hounsfield units (HU), area of HAS, and relative density (rHU) (HU clot/HU non-affected artery) were studied and related to time from symptom onset, clinical severity, stroke etiology, and laboratory parameters. A HAS was present in the middle cerebral artery (MCA) in 185 (7.2%) and further explored. The mean time from symptom onset to CT was 100 min (range 17–300). We found a time-dependent loss of density in the occluded M1 segment within the first 5 h (*N* = 118, 95% CI [−15, −2], *p* = 0.01). Further, the thrombus area in the M2 segment decreased with time (cubic trend *N* = 67, 95% CI [−63, −8], *p* = 0.02). Overall, and especially in the M2 segment, a lower clot area was associated with higher fibrinogen (−21.7%, 95% CI [−34.8, −5.8], *p* = 0.009). In conclusion, our results disclosed time-dependent changes of intracranial thrombi with regard to occlusion site, density and area.

## 1. Introduction

Acute occlusion of intracranial vessels is responsible for up to 80% of ischemic strokes [1,2]. The susceptibility of clot material to reperfusion therapy is being actively researched; however, more data is needed to fill the knowledge gap [3,4,5,6]. The composition of clots is thought to be dependent on the embolic source. Hence, fibrin-rich “white” thrombi are presumed to originate from high-flow larger arteries and thrombi with a predominant red blood cell (RBC) composition are more likely to stem from low-flow cardiac sources [3]. The clots composed predominantly of RBC are considered to be fresh, less compact and more hyperdense than fibrin-rich clots. After local plaque rupture in coronary vessels, the development of proximal and distal of fibrin-/thrombocyte-rich nidus have been reported [7]. A similar mechanism could take place for embolized clots in acute ischemic stroke (AIS). Indeed, interspersed formations of fibrin-platelet-rich deposits with linear collections of nucleated cells and erythrocytes have also been reported in AIS [4]. Several later studies, however, disclosed that the thrombi from these two locations do not differ in composition, being heterogeneous with both fibrin- and RBC-rich layers interspersed, probably reflecting time-dependent changes [4,8]. A recent CT densitometry study hypothesized that the clot loses its density by acquiring fibrin, since more hypodense clots were found in patients with lower fibrinogen values [9]. The RBC count, on the other hand, showed no correlation with clot density. In addition, the fibrinogen serum levels were lower in patients with larger intracranial clots.

In AIS, the hyperdense artery sign (HAS) on non-contrast-enhanced computed tomography (NECT) is thought to represent the intraluminal thrombus and subsequent arterial obstruction. A recent meta-analysis found a sensitivity and specificity of HAS for arterial obstruction on angiography of 52% and 95%, respectively [10]. Of note, thrombus characteristics can be evaluated reliably on non-contrast-enhanced CT by further characterizing the HAS [11,12]. In this regard, thrombi with lower Hounsfield units (HU) on NECT appear to be more resistant to pharmacological lysis and mechanical thrombectomy [11,13,14,15]. Given the varied choice of catheters and techniques currently available, pre-therapeutic thrombus characterization may help in the selection of the most effective method [16]. Here, we present the results of the retrospective three-center study on the time-dependent thrombus dynamics seen as HAS on NECT in patients with AIS.

## 2. Results

### 2.1. Patient Eligibility and Characteristics

**Figure 1:**
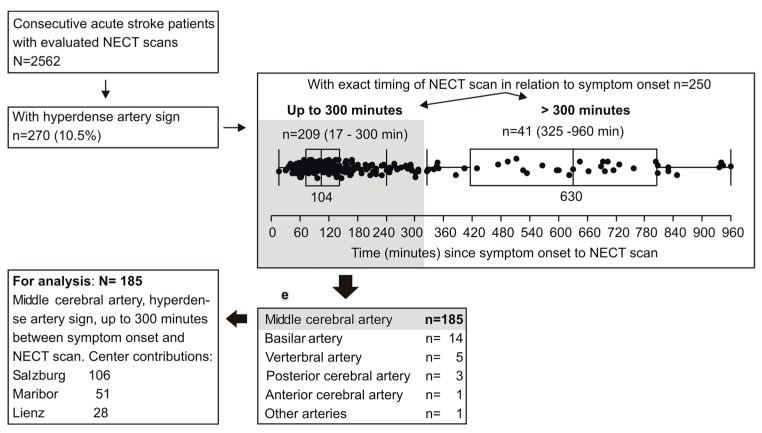
Flow of the patient selection process.

Of the 2562 patients with NECT scans, a HAS was present in 270 cases. Of those, 250 patients had the time between symptom onset and NECT scan exactly recorded: in 41 patients the elapsed time was >300 min (up to 960) (Figure 1). To avoid a potential bias arising from a lower number of patients beyond 300 min, only the patients with NECT performance within 300 min were selected for detailed analysis. Among the remaining 209, an occlusion of the middle cerebral artery (MCA) was by far prevailing (Figure 1).

To avoid a further bias due to low patient numbers for other occluded vessels, the present analysis was restricted to 185 patients with MCA involvement (Figure 1). Clinical details are depicted in Table 1 and give insight to characteristics of the entire cohort and in subgroups stratified for the affected MCA segment (proximal or distal). Patient subsets by MCA segment were fairly comparable in all aspects, except that thrombectomy was, by far, more frequently performed when proximal MCA was affected (Table 1).

### 2.2. Univariate Association between Timing of NECT (Non-Contrast-Enhanced CT) Relative to Symptom Onset and Ratio of Density (rHU) or Hyperdense Area

**Figure 2:**
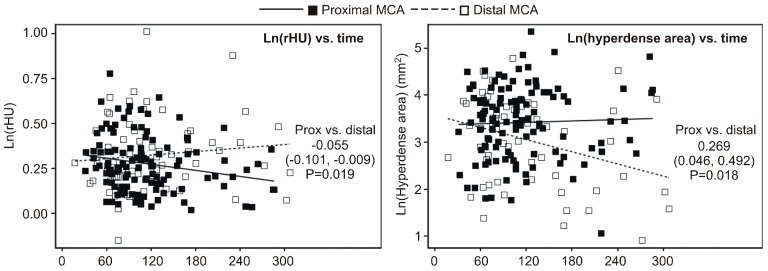
Exploration of the relationship between timing of NECT relative to symptom onset and ratio of density (rHU) (**left**) or hyperdense area (**right**). A separate linear mixed model (center as a cluster) was fitted to ln-transformed rHU and hyperdense area (to achieve normality of residuals) with time, MCA segment and time × MCA segment interaction term as independent variables. In the analysis of ln(rHU), there was no overall effect of time (*F* = 0.05, *p* = 0.824) and no overall difference between the MCA segments (*F* = 1.21, *p* = 0.274), but the interaction term was significant (*F* = 5.61, *p* = 0.019). Difference in slopes of ln(rHU) vs. time (per 60 min) at the two MCA segments is depicted numerically. Similarly, in the analysis of ln(hyperdense area), there was no overall effect of time (*F* = 1.27, *p* = 0.261) and no overall difference between the two MCA segments (*F* = 0.00, *p* = 0.964), but the interaction term was significant (*F* = 5.68, *p* = 0.018). Difference in slopes of ln(hyperdense area) vs. time (per 60 min) at the two MCA segments is depicted numerically.

Initial exploration of the relationship between the timing of NECT relative to symptom onset (time-lag) and the ratio of the density (rHU) or hyperdense area indicated that these relationships were apparently different at the proximal and distal MCA (Figure 2). In detail, rHU tended to decrease with a longer time-lag at the proximal MCA but not at the distal MCA, and the difference between the slopes of the two regression lines of ln(rHU) vs. time was significant (*p* = 0.019). In contrast, the hyperdense area tended to decrease with a longer time-lag at the distal MCA, but not at the proximal MCA. The difference between the slopes of the two regression lines of ln(hyperdense area) vs. time was significant (*p* = 0.018).

**Figure 3:**
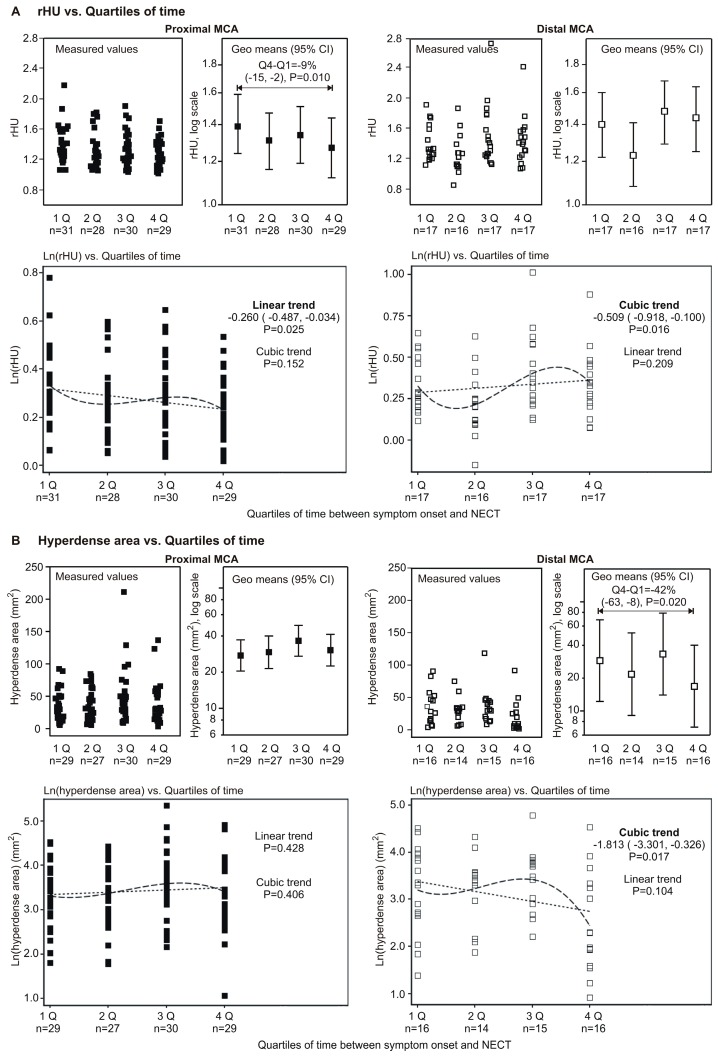
Univariate relationship between timing of NECT relative to symptom onset (as quartiles of time-lag) and ratio of density (rHU) (**A**) or hyperdense area (**B**) by segment of medial cerebral artery (MCA). (**A**) A mixed model (center as a cluster) was fitted to ln-transformed rHU (to achieve normality of residuals) with time-lag as the only independent variable. The relationship was tested for a linear, quadratic and cubic trend. At the proximal MCA, a significant linear decreasing trend was observed (depicted numerically) across quartiles of time-lag and values at the fourth quartile of elapsed time were by 9% lower than at the first quartile. At the distal MCA, values at the second quartile were lower than at the first quartile of the time-lag, and then increased to the third and fourth quartile, yielding a significant cubic trend (depicted numerically); however values at the fourth quartile of the time-lag were closely similar to the values at the first quartile; (**B**) The same analysis was repeated for the ln-transformed hyperdense area. At the proximal MCA, no apparent trend across quartiles of time was observed and values at the fourth quartile were closely similar to the values at the first quartile. At the distal MCA, values slightly increased towards the third quartile and then decreased to the fourth quartile of the time-lag, yielding a significant cubic trend (depicted numerically), and values at the fourth quartile were 42% lower than at the first quartile. Percentage difference between the fourth and the first quartile of time-lag = (1 − *e*^coeff^) × 100.

Since the sample was limited, and particularly for the MCA subset groups with inadequate power for an analysis based on an interaction term (MCA segment × time), we assessed the relationships of interest separately at the proximal MCA and the distal MCA. Moreover, we treated time as a categorical variable (by quartiles, see Table 1 for limit values) since cases for the later time-lag were relatively sparse and variable. The relationship between the rHU and hyperdense area vs. quartiles of time-lag is shown in Figure 3. The key findings were:

(a) rHU decreased linearly across quartiles of time (linear trend *p* = 0.025) at the proximal MCA and values at Q4 were 9% lower than at Q1 (*p* = 0.010) (Figure 1A); at the distal MCA, an apparent cubic trend (*p* = 0.016) was observed since rHU values declined from Q1 to Q2 and then increased at Q3 and Q4, and hence values at Q4 were actually no different than the values at Q1 (Figure 3A);
(b) At the proximal MCA, there was no apparent difference regarding the hyperdense area across quartiles of time (Figure 3B), whereas at distal MCA there was a significant cubic trend (*p* = 0.017)—the values slightly increased from Q1 to Q3, and then declined at Q4, so that the values at Q4 were 42% lower than at Q1 (*p* = 0.020) (Figure 3B).

### 2.3. Multivariate (Independent) Association between Timing of NECT Relative to Symptom Onset and Ratio of Density (rHU) or Hyperdense Area

With adjustment for age, we analyzed the clinical severity of disease at presentation (represented by the NIHSS score) and stroke etiology (TOAST criteria) categorized as “cardioembolic”, “large artery atherosclerosis” (two readily identifiable categories with specific, distinct pathophysiology) and “other” (unknown or undetermined) with different readouts of clot characteristics. The relationship between the timing of NECT relative to the symptom onset and rHU and hyperdense area remained practically unchanged (Table 2). Further findings were:

(a) At the proximal MCA, the rHU linearly decreased across quartiles of the time-lag (linear trend *p* = 0.019) and values at the fourth quartile were 10% lower than at the first quartile (*p* = 0.008); at the distal MCA, the cubic trend remained significant and there was no difference in rHU at the fourth vs. first quartile of the time-lag; and
(b) At the proximal MCA there was no apparent change in the hyperdense area across quartiles of the time-lag, whereas at the distal MCA the cubic trend remained significant and values at the fourth quartile were 39% lower than at the first quartile (Table 2).

### 2.4. Exploration of the Relationship between Serum Fibrinogen Levels and rHU or Hyperdense Area

**Figure 4:**
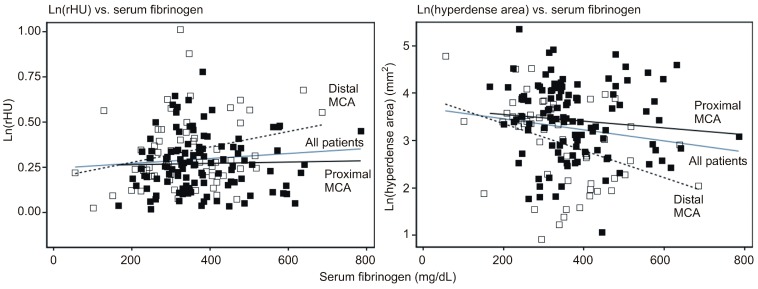
Adjusted regression lines of ln-transformed rHU (**left**) and hyperdense area (**right**) vs. serum fibrinogen, overall and by the segment of medial cerebral artery (MCA). Regressions are from the model depicted in Table 3.

On-admission serum fibrinogen levels were available for 170/185 patients (91.9%). Among these patients there were 111/118 (94.1%) with proximal MCA and 59/67 (88.1%) with distal MCA pathology. A separate mixed model (center as a cluster) was fitted to ln(rHU) and ln(hyperdense area) with the MCA segment, serum fibrinogen, stroke type (by TOAST criteria) and MCA segment × fibrinogen interaction term. Figure 4 depicts adjusted regressions of either dependent variable on the serum fibrinogen and adjusted estimates are shown in Table 3. In detail, we found:

(a) there was an overall trend of association between higher serum fibrinogen and higher rHU (2.3% higher with 100 mg/dL increase in fibrinogen). However, there was no association between fibrinogen and rHU in patients with an affected proximal MCA, whereas the association was stronger and statistically significant in patients with an affected distal MCA (4.2% higher rHU by 100 mg/dL increase in fibrinogen);
(b) for the entire cohort, higher fibrinogen was associated with a smaller hyperdense area (15% by 100 mg/dL increase in fibrinogen) (*p* = 0.005). However, this association was much weaker and not statistically significant in patients with an affected proximal MCA, whereas it was stronger and significant in patients with an affected distal MCA (Table 3). Due to incompleteness, data should be viewed with caution, but suggest that at proximal MCA, rHU apparently declines over the first 300 min after the stroke onset, though the hyperdense area does not appear to change. Further, neither of these two radiological outcomes seems to be associated with serum fibrinogen levels. Moreover, at the distal MCA, rHU does not appear to change while the hyperdense area tends to diminish over the first 300 min after the stroke onset. At the same time, a higher rHU and lower hyperdense area appear to be associated with higher serum fibrinogen.

## 3. Discussion

The efficacy of recanalization efforts in AIS is variable and biomarkers for stratifying patients with a lower probability of success are eagerly awaited. The anatomical site, composition and spread of clot in various arteries are potential parameters which could assist decision-making processes in order to individually optimize treatment [17,18]. NECT is a fast, widely available and readily used method in acute stroke and it enables non-invasive thrombus characterization. Importantly, thrombus characterization by NECT could provide additional useful information regarding clot susceptibility to thrombolysis and mechanical recanalization. Clot characteristics, however, could undergo dynamic changes over time as a multitude of biochemical pathways are activated when a vessel is occluded and the clot is exposed to hemodynamic and humoral changes of the local milieu [19,20,21]. These include a combination of proximal and distal apposition of new thrombotic material as well as proteolytic processes, which dissolve less compact thrombus material and leave a place for further fibrin meshwork propagation.

Here, we found indirect evidence of changes in clot composition and morphology within the first 5 h of AIS. Our study disclosed that MCA M1 clots, but not MCA M2 loses its density within the first 5 h. Moreover, we report a decline of the clot plane over time for the M2 segment. The area reduction was also associated with higher fibrinogen blood levels, which corroborates our previous observation [9]. The clots situated in the M1 segment did not change with regard to area and time, and thrombus characteristics did not correlate with fibrinogen levels.

Why is there a difference between the proximal and distal MCA occlusion with regard to time-dependent changes? This observation could, on one hand, be related to differences of embolic material, and indeed, large artery atherosclerosis was a more frequent etiology in M1 segment occlusion (16.9% vs. 4.5%). The other factor playing a possible role could be the availability of collateral perfusion regarding the occluded segment. This issue was not assessed in our study. Although there are conflicting reports, Kim et al. found higher proportions of RBCs and a lower proportion of fibrin in clots arising from cardioembolic (CE) than in those with large artery arteriosclerosis (LAA) etiology. The predominant histology of distal clots is not reported in published studies. Accordingly, we could speculate that distal thrombi, having originally more fibrin content that is more resistant to endogenous lysis, shield the RBC-rich part from degradation and accordingly these clots do not change in density with time. However, the later the patient arrives, the area of the distal thrombi seems to be somewhat smaller, possibly reflecting the degradation of RBC content later in time since the change is not obvious until the last quartile of time.

A time-dependent drop in clot density (absolute HU) was previously reported by Topcuoglu et al. [22]. In contrast to our findings, one study with 106 patients showed no changes in the relative HU density of the hyperdense artery within the first 4.5 h [23]. The reason for this discrepancy remains unclear. The density of the clot as seen on NECT is augmented by RBC content. Loss of density after embolism in MCA M1 occlusion is probably due to the preferential degradation of the erythrocyte-rich part of the clot [7]. Reports from a murine ischemic model provide evidence that the urokinase plasminogen activator is activated in the first 1 to 2 h following acute MCA occlusion, with gradual weakening of activity thereafter, which could explain our observation [19].

The second largest group concerning stroke pathogenesis consisted of patients with unknown etiology, accounting for 29%. Although we have not specifically reassessed details of the diagnostic stroke workup, the usual standard of care in stroke units includes blood analysis, 24 h electrocardiography (ECG), heart ultrasound and vessel imaging in every patient. Accordingly, it is unlikely that a lack of workup is responsible for this unexpectedly high number of cryptogenic strokes. With the presence of the intracranial clot, many of these patients could be classified as embolic stroke of undetermined source (ESUS) as the underlying etiology [24]. The prevalence of cryptogenic or ESUS strokes in cohorts with evaluation of the HAS is rarely reported. A pilot study identified nine patients with HAS of the MCA, which accounted for 20% of all stroke cases. Cryptogenic and ESUS stroke made up 26% of stroke cases in a larger study [25]. One study revealed that patients without detectable stroke etiology may have better clinical outcomes [26]. Further studies are required to prove whether hints for stroke etiology could be determined by analysis of clot morphology on NECT in cases where the causality remains unclear from ancillary investigations.

Limitations of our study are the usage of different scanning parameters and non-uniform slice thickness. This influences the detection rate of HA which is known to be dependent on slice thickness, and studies confirmed that thinner NECT slices have a greater sensitivity [12]. In addition, the rather low detection rate of HA in nearly 10% of all consecutive strokes could be interpreted as low sensitivity of our study. However, the prevalence of the HA sign among non-selected stroke patients and larger cohorts has not been reported so far. Selected populations, i.e., patients chosen for thrombolysis or with specific stroke syndromes such as posterior cerebral artery stroke, have higher detection rates, but this varies widely (5%–75%) [27]. In our study, some form of angiography (CT, magnetic resonance imaging (MRI) or digital subtraction angiography (DSA)) was performed in 63.8% patients with a correlation of vessel occlusion. For other patients, the clinical stroke syndrome was taken for the verification of vessel obstruction and this always corresponded to the side of the artery occlusion. Of note, we acknowledge the difficulty of discerning the hyperdense artery on plain CT without additional angiography but we presume that the combination of hematocrit correction and correlation with the clinical syndrome suffices for the identification of the occluded artery. Naturally, the presumption that time-dependent changes can be approximated from various patients is subjected to biases. Ideally, each patient should be re-examined with NECT at fixed time points. Such an approach, however, is not feasible due to ethical constraints and patient safety. Nevertheless, in our population, on follow up CT scans in 45 patients, HA was still visible (29%); of them 32 (71%) showed no change or had a drop in rHU values, thus further confirming our findings. Eventually, our observation needs to be confirmed in a larger cohort. Moreover, the characterization of thrombus dynamics beyond 5 h from symptom onset should follow as well.

## 4. Materials and Methods

We performed a retrospective study of consecutive patients with AIS who presented to the emergency department in three stroke centers. These were Christian-Doppler-Klinik Salzburg, Austria (CDK), University Clinical Center Maribor, Slovenia (MB) and Bezirkskrankenhaus Lienz, Austria (LZ). The study periods for CDK and MB were 2013–2015, and for LZ 2011–2014.

The inclusion criteria were age ≥18 years, presentation within 16 h from stroke onset and available head NECT. We excluded those cases were brain hemorrhage, brain tumor or non-stroke pathology was seen. In addition to usual laboratory examinations we recorded HbA1c and acute fibrinogen values. Stroke subtypes were classified according to the modified Trial of Org 10172 in Acute Treatment (TOAST) criteria [28]. NECT was performed before treatment with rt-PA in all patients.

CT scans were acquired in LZ and CDK with the multidetector CT scanner Sensation 64 (Siemens, Erlangen, Germany) and in MB with the multidetector CT scanner (Aquilion 64, Toshiba Medical Systems, Tochigi, Japan). The CT scans were reconstructed into 4, 2.4 and 3 mm (for CDK, LZ and MB, respectively) thick adjacent slices through the whole brain with the specifications of 120 kV (all centers) and 250, 440 and 150–350 mAs (for CDK, LZ and MB respectively) (mean value, using automatic exposure control) and matrix size of 512 × 512. The mean equivalent dose was 1.2 mSv for CDK and LZ and 1.9 mSv for MB.

The evaluation of CT-scans were performed by two experienced stroke physicians who were blinded to the clinical data. When in doubt of presence of hyperdense vessel sign, consensus was reached. HAS was recognized as the area of hyperattenuating artery on NECT. The hyperdense area was manually delineated in IMPAX software (Impax Laboratories Inc., Hayward, CA, USA), the vessel location and the side (when applicable) were recorded. Areas with calcifications (HU > 90) were not delineated. Measurements of hyperdense artery were made as previously described [11]. In short, the region of interest (ROI) was manually placed on the margins of the clot. Average HU was then obtained from all voxels within the ROI, summed across all slices (if present on more than one) producing HU sum. The final HU value was calculated by dividing the HU sum with number of slices. We recorded area in mm^2^ of manually circumscribed hyperdensity. The area was summed across slices (when present in more than one). Analyses depending on variables including time, disease severity and location of the HAS were performed with respective cohorts.

In order to correct for the impact of hematocrit values we measured the density of the vessel contralateral to affected one, in the case of basilar artery hyperdensity the measurement was in posterior cerebral artery. From measured final HU value, relative HU ratio (rHU) (final HU value divided by average HU of contralateral/appropriate non-affected artery) was derived. After hyperdense artery detection, the medical records were checked to ensure correspondence with clinical symptoms.

### Data Analysis

In line with the study objective, data analysis was conceived to explore the relationship, univariate and independent (adjusted), between time (defined as time elapsed between symptom onset and NECT scan, i.e., time-lag) and the two radiological outcomes—rHU and size of the hyperdense area. For all analyses, center was treated as a random effect to account for potential correlation of data coming from one site. In randomized trials, this approach was shown to improve power and maintain nominal coverage rates [29]. Univariate models contained “time” as the only fixed effect. Selection of covariates (additional fixed effects) in multivariate models was based on rationale that adjustments should account for biologically plausible potential confounders and/or moderators. We did not intend to detect “all possible effects” or to define the best set of explanatory variables of variability of rHU or hyperdense area. Finally, in an attempt to explore a potential biological background for the observed time effects on rHU and hyperdense area, we investigated the relationship between these outcomes and serum fibrinogen levels at admission. All mixed models were fitted using SAS for Windows 9.4 software (SAS Inc., Cary, NC, USA).

## 5. Conclusions

There are time-dependent changes in MCA thrombus morphology and composition within the first 5 h from symptom onset in patients with AIS. Moreover, we found that proximal and distal MCA clots differ with regard to these dynamics. Further studies, ideally with the evaluation of mechanically retrieved intracranial clots, are required to understand the complex pathophysiological processes determining the intrinsic and extrinsic post-processing of an intracranial thrombus.

## Figures and Tables

**Table 1:** Patient characteristics, overall and by the affected medial cerebral artery (MCA) segment. Data are median (range) or count (percent), unless otherwise stated.

Variables	All Patients		Proximal MCA		Distal MCA	
	*N*	Values	*N*	Values	*N*	Values
Age (years)	185	75 (19–98)	118	75 (41–97)	67	75 (19–98)
Men	185	82 (44.3)	118	52 (44.1)	67	30 (44.8)
Symptom onset to NECT (min)	185	100 (17–300) (Q1–Q3 = 71–136)	118	104 (31–286) (Q1–Q3 = 71–133)	67	94 (17–300) (Q1–Q3 = 70–147)
Side affected (left/right)	185	92 (49.7)/93	118	62 (52.5)/56	67	30 (44.8)/37
Average clot density (HU)	185	46.3 (36.1–56.1)	118	46.5 (36.9–56.1)	67	45.9 (36.1–55.3)
Non-affected side density (HU)	185	35.8 (18.6–45.7)	118	35.7 (24.4–45.7)	67	33.9 (18.6–45.7)
Ratio clot/non-affected rHU	185	1.30 (0.86–2.75)	118	1.30 (1.02–2.18)	67	1.32 (0.86–2.75)
Hyperdense area (mm^2^)	176	30.2 (2.5–211.4)	115	31.7 (2.9–211.4)	61	28.2 (2.5–119.0)
Admission NIHSS	185	16 (0–32)	118	17 (0–32)	67	13 (0–32)
TOAST class	185		118		67	
Cardioembolic		93 (50.3)		59 (50.0)		34 (50.8)
Large artery atherosclerosis		23 (12.4)		20 (16.9)		3 (4.5)
Other (all arterial dissections)		6 (3.2)		4 (3.4)		2 (3.0)
Undetermined		9 (4.9)		3 (2.5)		6 (8.9)
Unknown		54 (29.1)		32 (27.1)		22 (32.8)
Angiography performed *	185	118 (63.8)	118	89 (75.4)	67	29 (43.3)
Thrombolysis	185	139 (75.1)	118	90 (76.3)	67	49 (73.1)
Thrombectomy	185	52 (28.1)	118	48 (40.7)	67	4 (6.0)
Thrombolysis + thrombectomy	185	44 (23.8)	118	40 (33.9)	67	4 (6.0)
Usage of antiplatelets	185	55 (29.7)	118	33 (28.0)	67	22 (32.8)
Usage of anticoagulants	185	19 (10.3)	118	12 (10.2)	67	7 (10.5)
History of stroke or TIA	185	25 (13.5)	118	14 (11.9)	67	11 (16.4)
Peripheral arterial disease	185	14 (7.6)	118	9 (7.6)	67	5 (7.5)
Atrial fibrillation	185	91 (49.5)	118	56 (47.5)	67	35 (53.0)
Diabetes mellitus	185	31 (16.8)	118	19 (16.1)	67	12 (17.9)
Arterial hypertension	185	127 (68.6)	118	84 (71.2)	67	43 (64.2)
Carotid stenosis >50%	185	24 (13.0)	118	16 (13.6)	67	8 (11.9)
Chronic heart failure	185	30 (16.2)	118	21 (17.8)	67	9 (13.4)
Blood glucose (mg/dL)	184	119 (76–351)	118	119 (76–254)	66	120 (77–351)
Total cholesterol (mg/dL)	160	181 (78–300)	102	185 (78–300)	58	175 (99–275)
Serum fibrinogen (mg/dL)	170	346 (55–785)	111	350 (166–785)	59	335 (55–685)

* Computed tomography or/and magnetic resonance or/and digital subtraction angiography. HU—Hounsfield units; NECT—non-contrast enhanced computed tomography; NIHSS—National Institutes of Health Stroke Scale score; TIA—transitory ischemic attack; TOAST—trial of Org 10172 in acute stroke treatment criteria.

**Table 2:** Independent association between timing of NECT relative to the symptoms onset and ratio of density (rHU) or hyperdense area: summary of adjusted effects.

Associations	At Proximal MCA		At Distal MCA	
	Estimate (95% CI)	*p*	Estimate (95% CI)	*p*
*Dependent: rHU*				
Linear trend across time-lag quartiles	−0.280 (−0.513, −0.046)	0.019	0.230 (−0.224, 0.684)	0.315
Cubic trend across time-lag quartiles	−0.169 (−0.404, 0.066)	0.158	−0.480 (−0.914, −0.046)	0.031
Difference in fourth to first quartile (%)	−10 (−16, −3)	0.008	2 (−11, 17)	0.761
*Dependent: hyperdense area*				
Linear trend across time-lag quartiles	0.652 (−0.543, 1.848)	0.282	−0.976 (−2.586, 0.671)	0.243
Cubic trend across time-lag quartiles	−0.906 (−2.104, 0.292)	0.137	−2.092 (−3.672, −0.512)	0.011
Difference in fourth to first quartile (%)	11 (−24, 62)	0.581	−39 (−63, −1)	0.046

A separate mixed model (center as a cluster) was fitted to the ln-transformed rHU and hyperdense area (to achieve normality of residuals) with quartiles of time-lag, age, NIHSS score at admission and stroke etiology by TOAST criteria (categorized as “cardioembolic”, “large artery atherosclerosis” or “other”) as independents, and the linear, quadratic and cubic relationships between time and dependent variables were tested. Depicted are adjusted effects. Percentage difference between the fourth and the first quartile of time-lag = (1 − *e*^coeff^) × 100.

**Table 3:** Association between serum fibrinogen and ratio of density (rHU) or hyperdense area: summary of adjusted effects expressed as % change in dependent variable by 100 mg/dL increase in serum fibrinogen.

Affected Artery	rHU		Hyperdense Area (mm^2^)	
	Estimate (95% CI)	*p*	Estimate (95% CI)	*p*
Proximal and distal MCA	2.27% (−0.10, 4.67)	0.059	−15.2% (−24.3, −5.0)	0.005
Proximal MCA	0.41% (−2.23, 3.17)	0.769	−8.2% (−29.3, 4.5)	0.191
Distal MCA	4.16% (0.39, 8.08)	0.031	−21.7% (−34.8, −5.8)	0.009

A separate mixed model (center as a cluster) was fitted to the ln-transformed rHU and hyperdense area (to achieve normality of residuals) with quartiles of time-lag, stroke etiology by TOAST criteria (categorized as “cardioembolic”, “large artery atherosclerosis” or “other”), serum fibrinogen levels, MCA segment and fibrinogen × MCA segment interaction as independent variables. Percentage change in rHU or hyperdense area = (1 − *e*^coeff^) × 100.

## Abbreviations

AIS	Acute ischemic stroke
LAA	Large artery atherosclerosis
HAS	Hyperdense artery sign
NECT	Non-enhanced CT
TOAST	Trial of Org 10172 in Acute Treatment criteria
HU	Hounsfield units
rHU	Average HU of hyperdense artery/average HU of non-affected artery
DSA	Digital subtraction angiography
MRI	Magnetic resonance imaging
CT	Computertomography
ESUS	Embolic stroke of unknown source
CE	Cardioembolic
